# Bibliometric and visualization analysis of temporomandibular joint osteoarthritis from 2004 to 2024

**DOI:** 10.3389/froh.2025.1596551

**Published:** 2025-05-30

**Authors:** Chi Xu, Chongqing Yu, Tao Wang, Xiang Gao

**Affiliations:** ^1^ Department of Oral and Maxillofacial Surgery, Stomatological Hospital of Chongqing Medical University, Chongqing, China; ^2^ Chongqing Key Laboratory for Oral Diseases and Biomedical Sciences, Chongqing, China; ^3^ Chongqing Municipal Key Laboratory of Oral Biomedical Engineering of Higher Education, Chongqing, China

**Keywords:** temporomandibular joint, osteoarthritis, bibliometric, VOSviewer, CiteSpace

## Abstract

**Background:**

Temporomandibular joint osteoarthritis (TMJOA) is a severe condition affecting the temporomandibular joint, impairing essential oral functions such as chewing, speaking, and swallowing. Recent studies have made significant outcomes in pathogenesis, clinical symptoms, and therapy in the field of TMJOA. However, knowledge of research trends and hotspots of TMJOA is still lacking in this field. This study conducted a bibliometric analysis of TMJOA, providing a comprehensive overview of current research hotspots and trends.

**Methods:**

A total of 584 TMJOA-related records published between 2004 and 2024 were retrieved from the Web of Science Core Collection (WoSCC) database. VOSviewer, CiteSpace, the R package “bibliometrix”, and the Bibliometric website were used to analyze countries, institutions, journals, authors, and keywords to identify research trends in TMJOA.

**Results:**

Publications on TMJOA have shown a steady annual increase. Globally, China and the USA emerged as the leading contributors, accounting for over 60% of the publications. Sichuan University ranked first in total publications and citations, while the University of Northern Carolina led in average citations. The *Journal of Oral Rehabilitation* published the most studies, whereas the *Journal of Dental Research* had the highest impact factor (5.3) and H-index (158). Long X was the most prolific author, while Liu Y, Manfredini D, and Guarda-Narnini L were the top-cited authors with the highest H-indices. Keyword analysis revealed four primary research clusters: “pathogenesis mechanisms”, “clinical manifestations”, “regeneration research”, and “therapy research”.

**Conclusion:**

This bibliometric analysis highlights publication trends, research hotspots, citation patterns, and collaborative networks among countries, institutions, and authors in the TMJOA field. Future research is expected to focus on molecular signaling pathways and targeted therapies for TMJOA, with the ultimate goal of accelerating translational research to enhance clinical outcomes for patients.

## 1 Introduction

Temporomandibular joint osteoarthritis (TMJOA) is a chronic degenerative disease of the Temporomandibular joint (TMJ) and one of the most severe forms of Temporomandibular disorder (TMD). Globally, the prevalence of TMJOA is estimated to range from approximately 2% to 16% of the population (1, 2). The etiology of this degenerative joint disease is multifactorial and complex. As the only bilateral articulating joint in the human body, TMJ plays a critical role in essential physiological functions such as mastication, swallowing, breathing, and speaking (2). Risk factors for TMJOA include severe malocclusion, muscle overuse, and skeletal jaw asymmetry (3–5). As TMD progresses to TMJOA, irreversible changes occur in the condylar cartilage and subchondral bone. Early stages are characterized by cartilage degradation, subchondral bone loss, and increased angiogenesis, while late stages involve severe cartilage destruction, osteophyte formation, sclerosis, and cyst-like changes (6). These pathological changes often result in chronic pain, joint noises, and limited jaw movement.

To date, extensive research has been conducted to elucidate the pathogenesis of TMJOA. In the 2000s and 2010s, the imbalance between articular cartilage and mechanical loading was considered a key factor in disease progression (5, 7). However, recent studies have shown that mechanical overload alone cannot fully explain most TMJOA cases (3). Instead, inflammation has emerged as a critical contributor. Elevated levels of inflammatory cytokines such as Interleukin (IL)–12, IL-1β, IL-6 and tumor necrosis factor (TNF)–α have been detected in the synovial fluid (8, 9). Additionally, numerous studies have also emerged on other aspects, such as chondrocyte apoptosis (10–14), catabolic enzymes (15), and subchondral bone remodeling (16–19).

Furthermore, treatment strategies for TMJOA have advanced significantly. Traditional approaches, such as physical therapy, occlusal splints, nonsteroidal anti-inflammatory drugs (NSAIDs), arthrocentesis with lubrication or corticosteroids, and surgical interventions, effectively alleviate symptoms and moderately slow disease progression (20). Recent innovations, such as drug delivery systems (21, 22) and tissue engineering therapies (23–25), aim to restore TMJ tissues, reflecting a growing interest in TMJOA. Despite these advancements, a limited number of studies have investigated the research and publication trends of TMJOA in the Web of Science. To address this gap, our study conducts a comprehensive bibliometric analysis of TMJOA literature from 2004 to 2024, employing visualization tools to identify key trends and predict future research directions.

## 2 Materials and methods

### 2.1 Data source and search strategy

The Web of Science Core Collection (WoSCC), a globally recognized database platform by Clarivate Analytics, was selected for its authoritative coverage of over 12,000 international academic journals (26). Therefore, according to previous studies, we chose it to obtain global academic information for bibliometric analysis (27, 28). We extracted all the published literature from WoSCC, and the search date was from 1 January 2004 to 31 December 2024. In present study, the detailed search strategy was as follows: (TS = (“TMJOA”) OR TS = (“Temporomandibular joint osteoarthritis”) OR TS = (“TMJ osteoarthritis”) OR TS = (“osteoarthritis of temporomandibular joint”) OR TS = (“osteoarthritis of the temporomandibular joint”) OR TS = (“OA of TMJ”) OR TS = (“osteoarthritis of TMJ”) OR TS = (“OA of temporomandibular joint”) OR TS = (“TMJ osteoarthrosis”)) AND Document types = (ARTICLE OR REVIEW) AND Language = (English). Using the Web of Science filter tool to remove other types of publications, 584 publications were selected, and all valid data, including publishing year, title, author names, nationalities, affiliations, abstract, keywords, H-index, and name of journals, were exported in TXT format. Ethical approval was not required as no animals or humans were involved in the study.

### 2.2 Bibliometric analysis and visualization

We used VOSviewer 1.6.20, CiteSpace 6.4.R1, R package “Bibliometrix” software, and the Bibliometrics website for bibliometric analysis. Specifically, VOSviewer was performed to analyze the co-authorship, co-citation, and co-occurrence in detail. In addition, CiteSpace, developed by Professor Chen C (Drexel University, USA), was used to build a dual-map overlay for journals, cluster analysis of co-citation, and detection of references and intense citation bursts. Moreover, the R package “bibliometrix” software (biblioshiny) was used in this study to visualize publications output across countries, authors, and journals, as well as to generate a three-field plot analysis.

## 3 Results

### 3.1 Overview and annual publication

**Figure 1:**
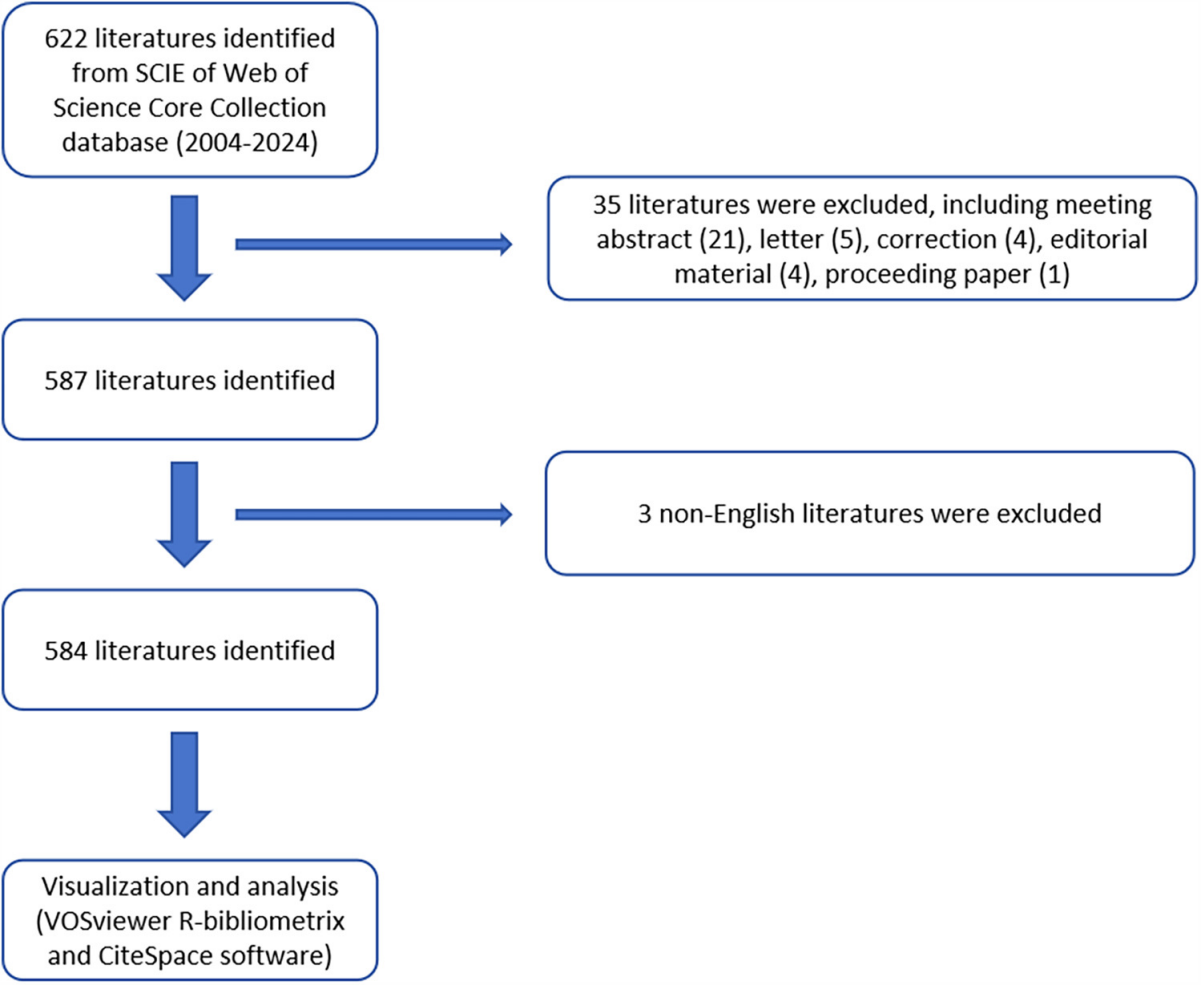
Flowchart of the screening process.

**Figure 2:**
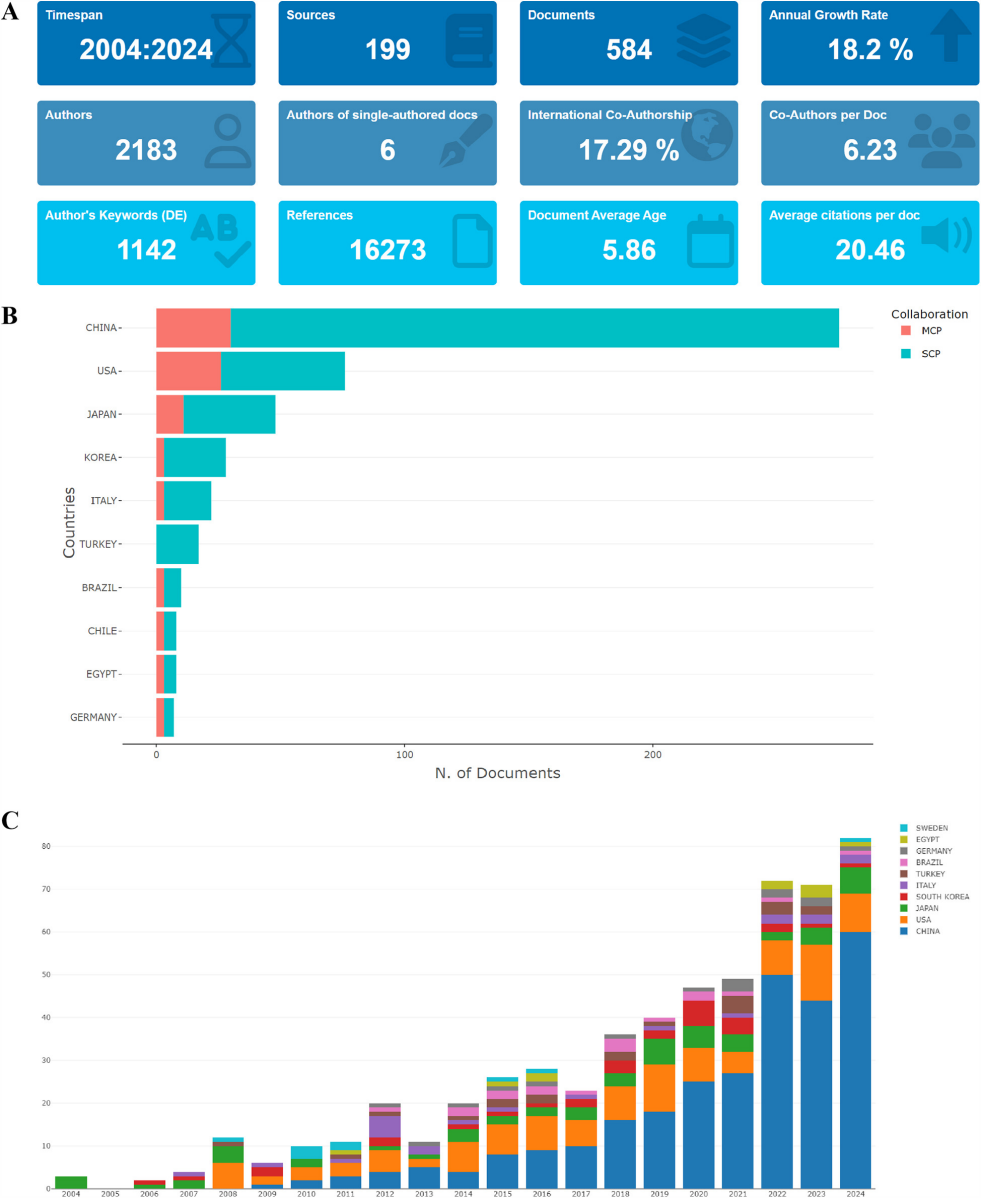
**(A)** General information of articles related to TMJOA in this study; **(B)** the total number of publications in the top 10 most productive countries; **(C)** the annual number of publications in the top 10 most productive countries from 2004 to 2024.

According to the search strategy, 622 literatures were identified from 2004 to 2024. Subsequently, 587 literatures were identified by excluding the meeting abstract (21), letter (5), correction (4), editorial material (4), and proceedings papers (1). Eventually, 584 literatures were identified by excluding 3 non-English literatures (Figure 1). The leading information was described in Figure 2A, using the “biblioshiny”. The analysis revealed that these 584 articles involved 2,183 authors and were published across 199 journals. We also found that the annual growth rate of publications in this field was 18.2%. The international co-authorship accounted for 17.29%. Besides, there were a total of 16,273 references and 1,142 keywords in the field of TMJOA. The average age of articles was 5.86, while the average number of citations per article was 20.46. The results indicate that TMJOA research has demonstrated remarkable growth over the past two decades, and this field features an active international collaboration network.

Figure 2B presents the total number of publications from each identified country and the collaboration patterns. China published the most papers (275, 47.1%), followed by the USA (76, 13.0%), Japan (48, 8.2%), Korea (28, 4.8%) and Italy (22, 3.8%). We also found that the Multiple Country Publication (MCP) of the top three countries were 30 (10.91%), 26 (34.21%), and 11 (22.92%), respectively, indicating their active role in promoting international collaboration. As illustrated in Figure 2C**,** the general trend of the number of publications in the field of TMJOA was an increase. The annual publications of the top 10 countries/regions increased by 79 from 2004 (3) to 2024 (82). Furthermore, the number of publications from 2022 to 2024 was 225 (38.5% of total publications), indicating that TMJOA research has increasingly become a focal point for researchers and has entered a phase of rapid development.

### 3.2 Analysis of countries

**Figure 3:**
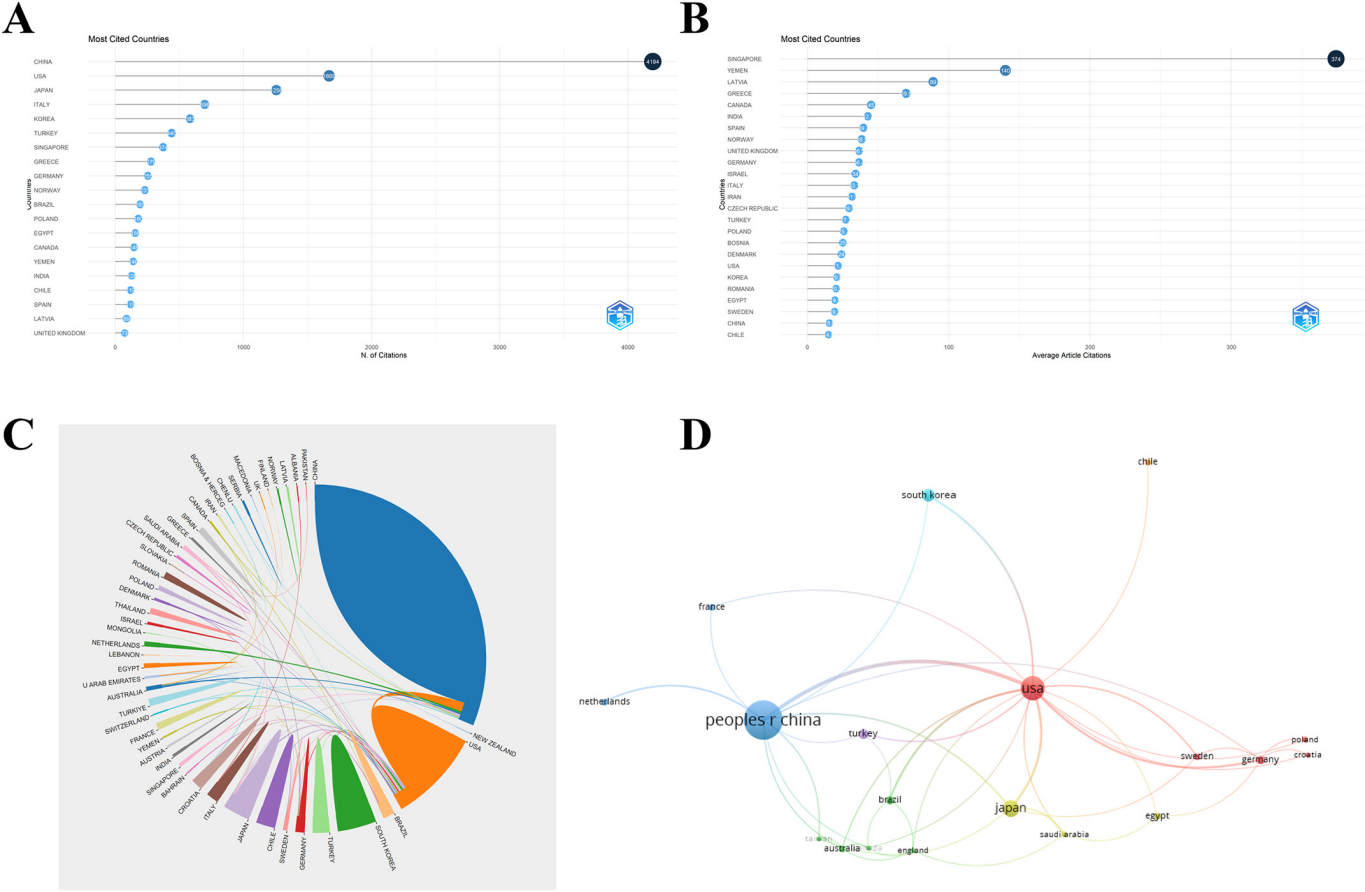
**(A)** The top 20 countries of total citations related to TMJOA. **(B)** The top 25 countries of average article citations related to TMJOA. **(C)** Global collaboration on the topic of TMJOA from 2004 to 2024. **(D)** Visualization map of international cooperation between countries in the field of TMJOA.

Figure 3A and Table 1 illustrate that publications from China had the highest total citation frequencies (4,194). The USA ranked second with a number of 1,669, followed by Japan (1,256), Italy (698) and Korea (583). Regarding the average article citations (Figure 3B), Singapore had the highest average citation frequencies (374). Yemen ranked second with a number of 140, prior to Latvia (89), Greece (69.8), and Canada (45). In respect of the global collaboration analysis, Figures 3C,D showed that China exhibited the highest output volume and worked closely with the USA, Netherlands, Australia, and Singapore.

**Table 1:** Global research output on temporomandibular joint osteoarthritis (TMJOA): Top 10 contributing countries by publication volume.

Rank	Country	Publications (%)	Citations
1	China	275 (47.1%)	4,194
2	USA	76 (13.0%)	1,669
3	Japan	48 (8.2%)	1,256
4	Korea	28 (4.8%)	583
5	Italy	22 (3.8%)	698
6	Turkey	17 (2.9%)	440
7	Brazil	10 (1.7%)	193
8	Chile	8 (1.4%)	119
9	Egypt	8 (1.4%)	156
10	Germany	7 (1.2%)	255

### 3.3 Analysis of institutions and authors

**Figure 4:**
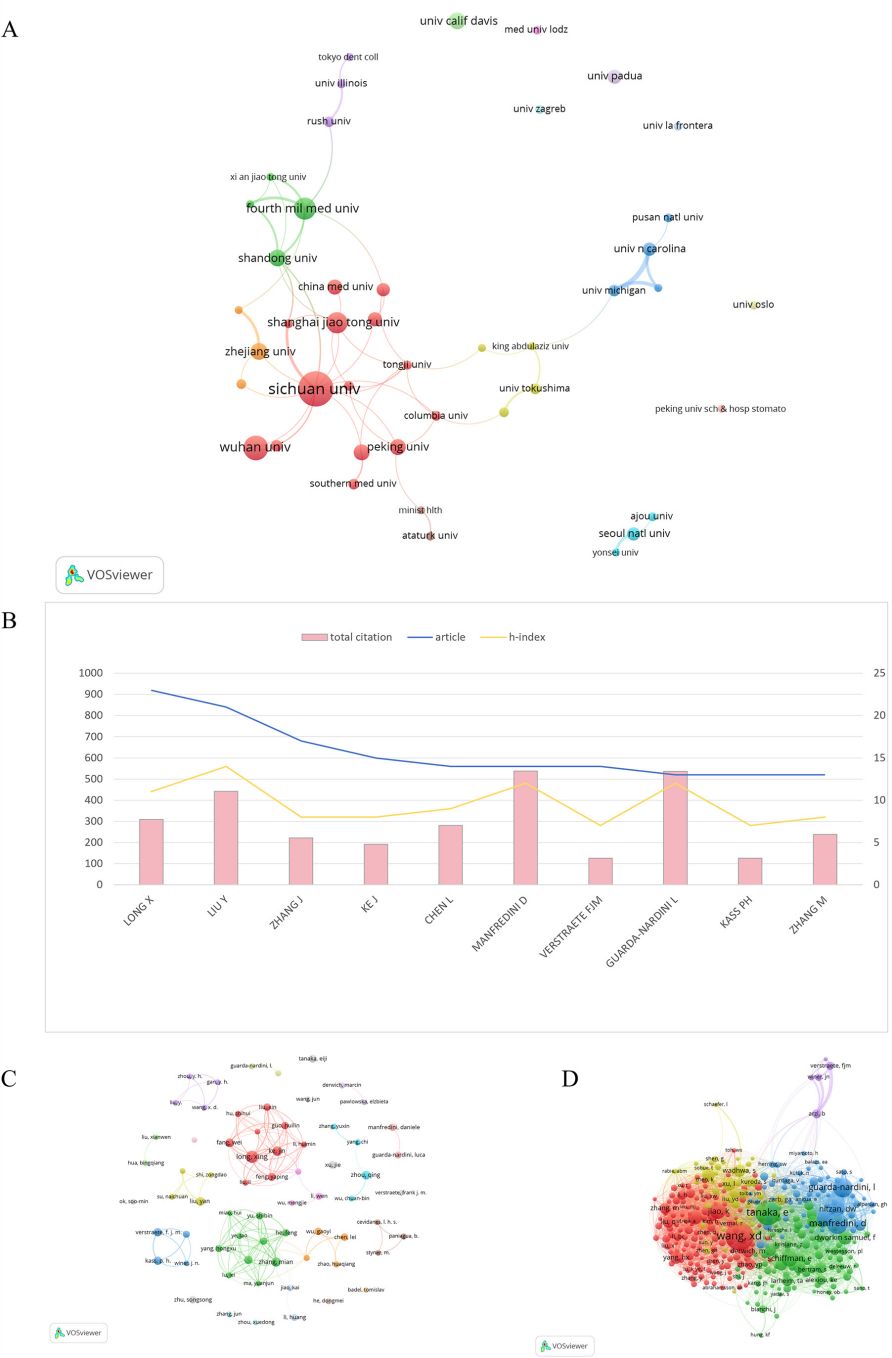
**(A)** Co-authorship analysis of institutions in the field of TMJOA. **(B)** The top 10 prolific authors in the field of TMJOA. **(C)** Collaboration analysis of authors on the topic of TMJOA from 2004 to 2024. **(D)** Visualization map of co-cited authors in the field of TMJOA.

A ranking of the top 10 contributing institutions based on publication output is presented in Table 2. The first was Sichuan University (145 publications), followed by Wuhan University (81 publications), Fourth Military Medical University (69 publications), and the University of California Davis (39 publications). Concerning the number of citations, Fourth Military Medical University ranked first for total citations, whereas the University of North Carolina ranked first for average citations. Figure 4A exhibited the network visualization of collaboration between institutions, which showed that there was a strong cooperation relationship between institutions such as Sichuan University, Shanghai Jiao Tong University, Fourth Military Medical University, and Shandong University in China and the University of North Carolina, University of Michigan in the USA.

**Table 2:** Leading 10 institutions by publication output in temporomandibular joint osteoarthritis (TMJOA) research.

Rank	Institutions	Article counts	Total number of citations	Average number of citations	Total number of first authors	Total number of first author citations	Average number of first author citations
1	Sichuan Univ	145	565	3.9	45	214	4.76
2	Wuhan Univ	81	328	4.05	29	112	3.86
3	Fourth Mil Med Univ	69	524	7.59	18	134	7.44
4	Univ Calif Davis	39	160	4.1	16	68	4.25
5	Peking Univ	36	357	9.92	12	92	7.67
6	Zhejiang Univ	35	82	2.34	13	50	3.85
7	Shanghai Jiao Tong Univ	33	87	2.64	14	34	2.43
8	Univ N Carolina	32	388	12.13	3	35	11.67
9	Shandong Univ	28	145	5.18	11	69	6.27
10	Univ Michigan	27	171	6.33	7	65	9.29

In this study, we identified 2,183 authors in the field of TMJOA in total. The top 10 prolific authors were analyzed, along with the total citations and h-index. Figure 4B illustrates that Long X had the most publications (23), followed by Liu Y (21) and Zhang J (23). Regarding the h-index, LIU Y, MANFREDINI D, and GUARDA-NARDINI L ranked in the top 3 in the field of TMJOA; in addition, the three of them were also among the top three in total citation numbers.

VOSviewer also visualized the network between authors, as shown in Figure 4C. Authors from the same country collaborated more frequently and had strong connections. The co-citation analysis considered the relatedness of the items based on the numbers they were co-cited. Figure 4D illustrated that 330 authors with a minimum of 10 documents were analyzed using VOSviewer. The top 5 authors with the largest total link strength were as follows: Wang, XD (total link strength = 3,751 times), Tanaka, E (total link strength = 3,186 times), Jiao, K (total link strength = 2,240 times), Manfredini, D (total link strength = 2,186 times), and Guarda-Nardini, L (total link strength = 2,132 times).

### 3.4 Analysis of journals and research fields

**Figure 5:**
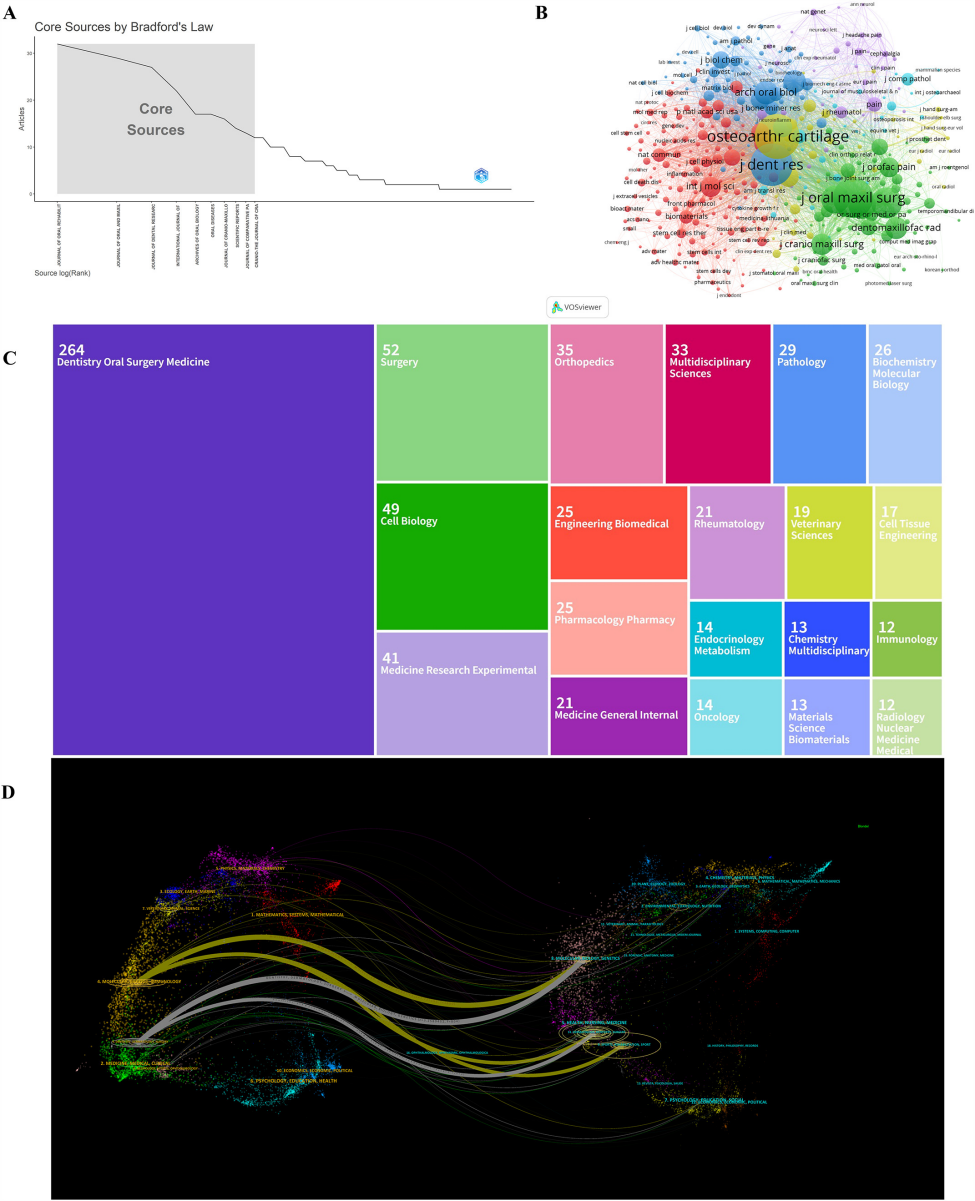
**(A)** Core journals in the field of TMJOA. **(B)** Visualization of journals that were co-cited in more than 10 citations. **(C)** Treemap of top 20 research fields on the topic of TMJOA. **(D)** The dual-map overlay of journals in the field of TMJOA.

We presented the publication counts, impact factors (IF), and H-index values of the top 10 most productive journals involved in this study, as detailed in Table 3. The *Journal of Oral Rehabilitation* (impact facto*r* = 3.1, 2024) ranked first with 32 publications. There were 29 publications in the *Journal of Oral and Maxillofacial Surgery* (IF = 2.3, 2024), 27 publications in the *Journal of Dental Research* (IF = 5.7, 2024), 22 publications in the *International Journal of Oral and Maxillofacial Surgery* (IF = 2.2, 2024) and 17 articles in *Archives of Oral Biology* (IF = 2.2, 2024). Figure 5A illustrates the core sources identified through Bradford's Law, utilizing the biblioshiny. The core zone comprised 10 journals, which collectively published 199 articles, accounting for 34.08% of the total 584 articles in the field. In addition, a co-citation analysis of journals was conducted by VOSviewer, with journals having a minimum of 10 citations included in the analysis. As shown in Figure 5B, a total of 414 journals were represented based on their link strength. The top 5 journals with most total link strength are as follows: Osteoarthritis and Cartilage (total link strength = 57,167 times), *Journal of Dental Research* (total link strength = 50,542 times), *Journal of Oral and Maxillofacial Surgery* (total link strength = 33,597 times), Archives of Oral Biology (total link strength = 23,472 times), and Journal of Oral Rehabilitation (total link strength = 20,581 times).

**Table 3:** Top 10 most prolific journals in TMJOA research: productivity and scholarly impact.

Rank	Journal	Count	Percentage %	IF	H-Index
1	JOURNAL OF ORAL REHABILITATION	32	5.479452055	3.1	84
2	JOURNAL OF ORAL AND MAXILLOFACIAL SURGERY	29	4.965753425	2.3	109
3	JOURNAL OF DENTAL RESEARCH	27	4.623287671	5.7	158
4	INTERNATIONAL JOURNAL OF ORAL AND MAXILLOFACIAL SURGERY	22	3.767123288	2.2	90
5	ARCHIVES OF ORAL BIOLOGY	17	2.910958904	2.2	79
6	ORAL DISEASES	17	2.910958904	2.9	77
7	JOURNAL OF CRANIO-MAXILLOFACIAL SURGERY	16	2.739726027	2.1	67
8	SCIENTIFIC REPORTS	14	2.397260274	3.8	149
9	JOURNAL OF COMPARATIVE PATHOLOGY	13	2.226027397	0.8	65
10	CRANIO-THE JOURNAL OF CRANIOMANDIBULAR & SLEEP PRACTICE	12	2.054794521	2	40

Besides, a treemap diagram was employed to visually present the top 20 research fields, as shown in Figure 5C. In detail, the most prevalent research fields were dentistry oral surgery medicine, surgery, cell biology, medicine research experimental, and orthopedics. Figure 5D depicts five primary citation paths marked in yellow and grey. The two main paths revealed that documents published in the field of molecular, biology, and genetics were predominantly cited by researchers from molecular, biology, and immunology as well as dentistry, dermatology, and surgery. The remaining three citation pathways showed that articles published in sports, rehabilitation, and sport, as well as dermatology, dentistry, and surgery, were mainly cited by documents in molecular, biology, and immunology, as well as dentistry, dermatology, and surgery.

### 3.5 Analysis of citation and co-citation

**Figure 6:**
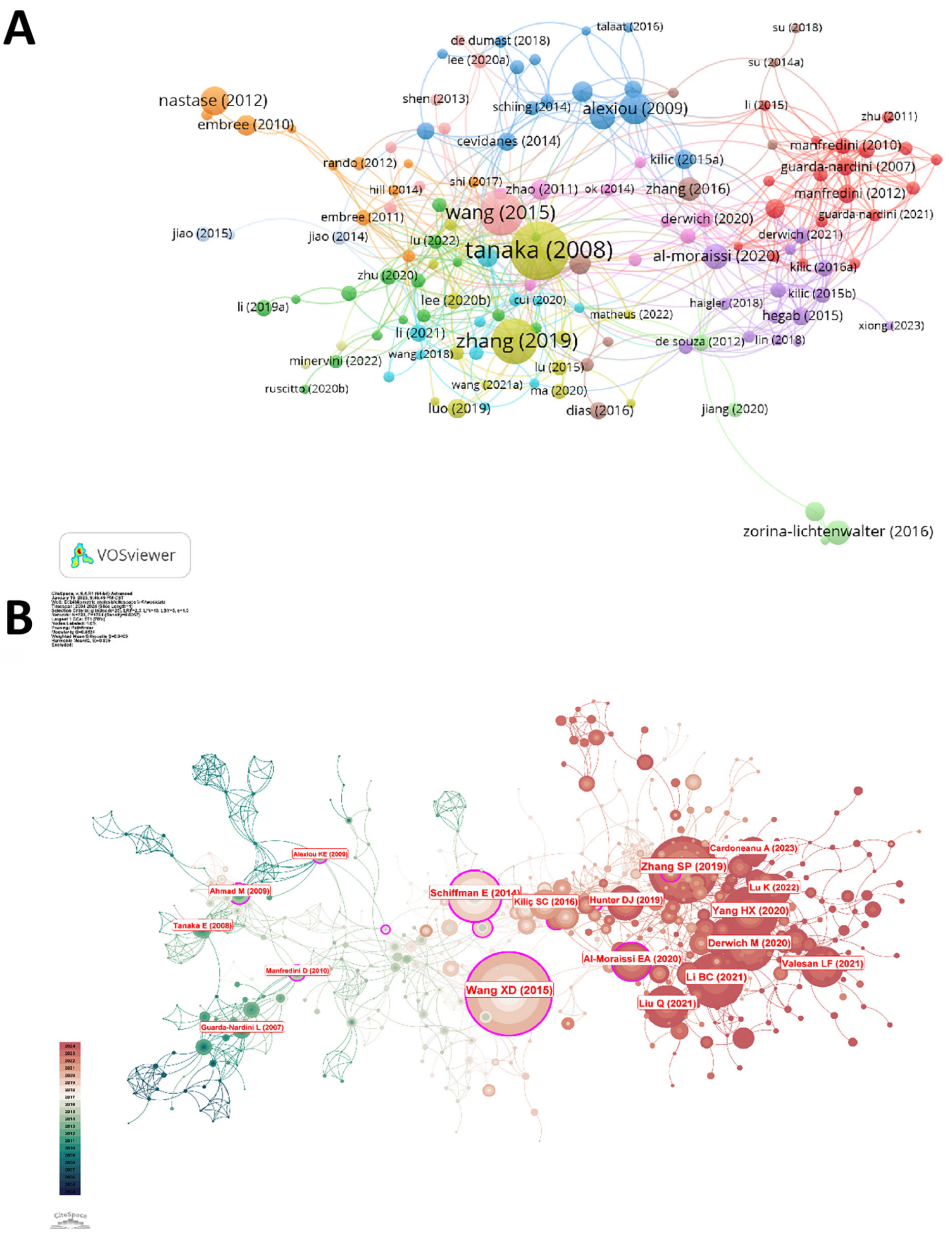
**(A)** Network map of citations analysis of articles in TMJOA. **(B)** Visualization of co-citation analysis of references.

As presented in Figure 6A, 144 articles in the field of TMJOA had over 25 citations. Table 4 shows the top 10 most cited articles. There were 579 citations for “Degenerative disorders of the temporomandibular joint: etiology, diagnosis, and treatment”, followed by “MSC exosomes alleviate temporomandibular joint osteoarthritis by attenuating inflammation and restoring matrix homeostasis”, with 374 citations. The third-ranked document was “Current understanding of pathogenesis and treatment of TMJ osteoarthritis”, with 339 citations.

**Table 4:** The 10 most highly cited publications with general information in TMJOA research.

Rank	Title	Corresponding author	Journal	IF	Publication year	Total citations
1	Degenerative disorders of the temporomandibular joint: etiology, diagnosis, and treatment	L G Mercuri	Journal of Dental Research	5.7	2008	579
2	MSC exosomes alleviate temporomandibular joint osteoarthritis by attenuating inflammation and restoring matrix homeostasis	Wei Seong Toh	Biomaterials	12.8	2019	374
3	Current understanding of pathogenesis and treatment of TMJ osteoarthritis	Y H Zhou	Journal of Dental Research	5.7	2015	339
4	Evaluation of the severity of temporomandibular joint osteoarthritic changes related to age using cone beam computed tomography	K Tsiklakis	Dentomaxillofacial Radiology	2.9	2009	194
5	Biglycan: a multivalent proteoglycan providing structure and signals	Liliana Schaefer	Journal of Histochemistry & Cytochemistry	1.9	2012	167
6	The hierarchy of different treatments for arthrogenous temporomandibular disorders: A network meta-analysis of randomized clinical trials	Andreas Neff	Journal of Craniomaxillofacial Surgery	2.1	2020	140
7	Quantification of condylar resorption in temporomandibular joint osteoarthritis	C Phillips	Oral Surgery, Oral medicine, Oral pathology, Oral radiology, and endodontics	N/A	2010	131
8	Genetic predictors of human chronic pain conditions	L Diatchenko	Neuroscience	2.9	2016	129
9	Upregulation of lncRNA HOTAIR contributes to IL-1β-induced MMP overexpression and chondrocytes apoptosis in temporomandibular joint osteoarthritis	Zhenlin Wang	Gene	2.6	2016	115
10	Expression of proinflammatory cytokines in osteoarthritis of the temporomandibular joint	Mariano Sanz	Archives of Oral Biology	2.2	2008	98

**Figure 7:**
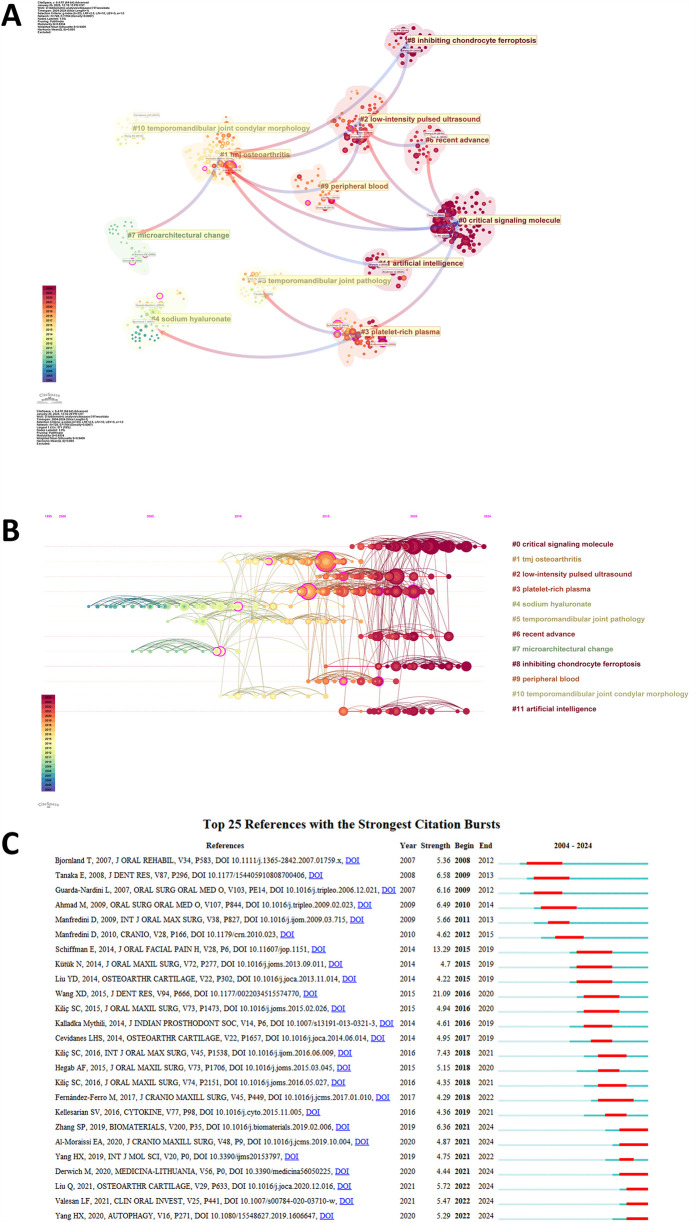
**(A)** Cluster dependencies of co-citations in the field of TMJOA. **(B)** Timeline of the cluster co-citations analysis of references. **(C)** Top 25 references with strongest citation bursts of publications related to TMJOA.

Moreover, we used Citespace to program a co-citation analysis (Figure 6B) to show the most influential literature. Figures 7A,B showed that the co-cited documents were assigned to 12 leading cluster labels as follows: critical signaling molecular; TMJ osteoarthritis; low-intensity pulsed ultrasound; platelet-rich plasma; sodium hyaluronate; temporomandibular joint pathology; recent advances; microarchitectural change; inhibiting chondrocyte ferroptosis; peripheral blood; temporomandibular joint condylar morphology; artificial intelligence. Upon further analysis, these 12 clusters revealed three dominant research trends. The first trend centered on pathogenic mechanisms underlying TMJOA (#0, #1, #5, #7, #8, #10). The second trend concerned research on novel treatment strategies for TMJOA (#2, #3, #4, #9), while the third focused on artificial intelligence and medicine (#6, #11). In addition, this visualization illustrated the evolution of research trends and the interdependent relationships among the various clusters.

Citation burst serves as a significant indicator, reflecting the references that attract heightened interest from researchers within a specific domain over a defined period (29). We used CiteSpace to identify the top 25 references with the strongest citation bursts and presented the results in Figure 7C. The article “Current understanding of pathogenesis and treatment of TMJ osteoarthritis”, published in 2008, ranked first in strength (21.09).

### 3.6 Analysis of keywords and hotspots

**Figure 8:**
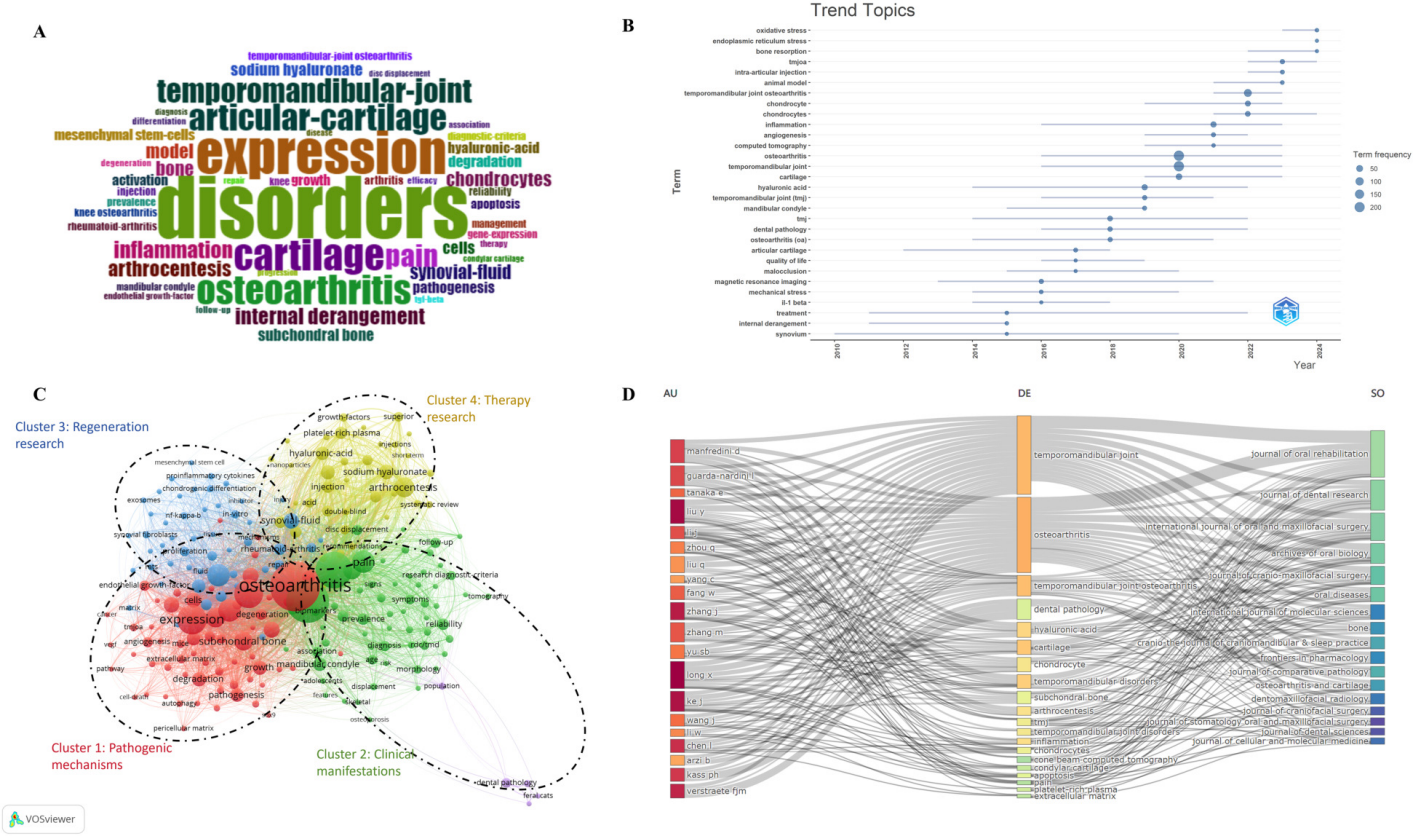
**(A)** Word cloud analysis of keywords in the field of TMJOA. **(B)** Trend topic analysis of keywords. **(C)** Network visualization of co-occurrence analysis of keywords on the topic of TMJOA. **(D)** Three-field plot of the keywords analysis on TMJOA (middle field: keywords; left field: authors; right field: journals).

Keyword analysis plays a pivotal role for researchers in identifying emerging trends and key research hotspots in TMJOA. To conduct the keyword analysis, we performed a word cloud analysis using the “biblioshiny” tool to assess the frequency and prominence of various keywords. As shown in Figure 8A, “disorders” and “expression” were the two most prevalent keywords. Additionally, we also used “biblioshiny” for trend topic analysis and identified the top 30 keywords with the highest burst strength, as shown in Figure 8B. In 2010, the keyword “synovium” emerged and remained prevalent until 2020. During this period, additional keywords related to etiology and risk factors also surfaced, including “malocclusion”, “mechanical stress”, and “IL-1 beta”. In recent years, the focus of research has shifted towards the pathogenic mechanisms of TMJOA, with keywords such as “oxidative stress” and “endoplasmic reticulum stress” gaining prominence.

In bibliometrics, keyword co-occurrence analysis is widely used to identify emerging research topics and trends. We conducted this analysis using VOSviewer, with keywords defined as those occurring over 5 times in the titles or abstracts of all selected papers. After eliminating duplicates, the results are presented in Figure 8C. The 197 identified keywords were mainly classified into 4 clusters as follows: Cluster 1: pathogenic mechanisms (red); Cluster 2: clinical manifestations (green); Cluster 3: regeneration research (blue); Cluster 4: therapy research (yellow). These results exhibited the most prominent research topics in TMJOA so far. The primary keywords in the “pathogenic mechanisms” cluster were expression, degeneration, subchondral bone, and angiogenesis. The keywords frequently used for the “clinical manifestations” cluster were internal derangement, biomarkers, pain, and disorders. In the “regeneration research” cluster, the principal keywords were synovial fluid, proliferation, inflammation, and repair. The keywords used most in the “therapy research” cluster were arthrocentesis, sodium hyaluronate and platelet-rich plasma. The results revealed that the most prominent areas of TMJOA research encompassed the 4 directions mentioned above.

Figure 8D represented a three-field graph generated using “biblioshiny”, associating authors, keywords, and journals. It was possible to observe the links between the principal elements through this, and their relationship was exhibited directly by the strength of the connection links (30). The most frequent keywords were “Temporomandibular joint”, “osteoarthritis”, “dental pathology”, “hyaluronic acid”, and “cartilage”. The authors Kass PH and Verstraete FJM were strongly connected with the keywords “dental pathology” and “Temporomandibular joint”, establishing the relatively strongest links. In turn, it can be found that the heaviest links were related to the Journal of Oral Rehabilitation, and it also covered most of the papers related to the keywords “Temporomandibular joint” and “osteoarthritis”. Moreover, the keyword “cartilage” was strongly connected with the Journal of Dental Research. Therefore, this visualization suggested that as the primary site of pathology in temporomandibular joint osteoarthritis (TMJOA), cartilage remained the central focus of current research in this field.

## 4 Discussion

### 4.1 General information

This bibliometric analysis identified key trends in temporomandibular joint osteoarthritis (TMJOA) research within the Web of Science Core Collection (WoSCC) from 2004 to 2024. The 2004–2024 timeframe was selected based on our preliminary bibliometric analysis that revealed a notable publication surge in 2008, with annual output tripling compared to the previous year (12 publications vs. 4 publications), indicating markedly increased research attention in TMJOA. Following a six-year stabilization period, the field has demonstrated consistent growth since 2014. This developmental trajectory motivated our systematic investigation of research trends spanning the two-decade period from 2004 to 2024. Annual publications increased steadily from 2004 to 2024 (Figure 2). Globally, 52 countries contributed to TMJOA studies, with China leading in total publications (275, 47.1%), followed by the USA (76, 13.0%), Japan (48, 8.2%), South Korea (28, 4.8%), and Italy (22, 3.8%). While China ranked first in both publications and total citations, its lower average citation count suggests room for improving research impact. Notably, Singapore achieved the highest average citations (374), surpassing Yemen (140) and Latvia (89), underscoring their significant influence despite fewer publications.

Our analysis revealed that China and the USA emerged as the leading contributors to TMJOA research, collectively dominating both authorship and institutional output (Table 1). Similarly, the top 10 institutions were exclusively based in these two countries, underscoring their pivotal role in advancing the field. As illustrated in Figure 4B, Liu Y, Manfredini D, and Guarda-Nardini L ranked highest in citation frequency and H-indices, reflecting their global recognition and scholarly impact. However, collaboration networks remained limited, with partnerships primarily confined to intra-institutional efforts and minimal cross-border engagement (Figure 4C). In the future, researchers from various countries and institutions should strengthen their collaboration to improve the research of TMJOA.

### 4.2 Research hotspots and trends

Our study constructed a co-occurrence network of keywords based on the frequency of keyword occurrence in the titles and abstracts of all included publications. Figure 8C showed 4 main clusters, each representing a distinct research trend: pathogenic mechanisms (red), clinical manifestations (green), regeneration research (blue), and therapy research (yellow). These clusters highlight current research priorities and provide a foundation for predicting future trajectories.

#### 4.2.1 Pathogenic mechanisms

Co-occurrence analysis of keywords identified “expression”, “subchondral bone”, “degeneration”, and “angiogenesis” as critical research hotspots warranting further investigation. Alteration of subchondral bone is a prominent feature of TMJOA, occurring in both early and late stages. The balance between osteogenesis and osteoclastogenesis plays a pivotal role in this process. Excessive subchondral bone loss is a hallmark of early-stage TMJOA, as demonstrated in various animal models induced by chemical, surgical, mechanical, or genetic methods. These models consistently show trabecular bone loss and reduced bone mineral density (BMD) (3, 31). The receptor activator of nuclear factor-*κ*B ligand (RANKL)/osteoprotegerin (OPG) system is a key signaling pathway in this process. Chondrocytes in degraded cartilage upregulate osteoclastogenesis in the subchondral bone, disrupting the balance between bone formation and resorption (17). Additionally, the RANTES-chemokine receptors-Akt2 (RANTES-CCRs-Akt2) axis has been identified as another mediator of early-stage subchondral bone loss (32). In late-stage TMJOA, bone mass increases, but the mechanical structure deteriorates, accompanied by reduced BMD, osteophyte formation, and sclerosis (33). Notably, angiogenesis also plays a crucial role in TMJOA progression. Specifically, increased angiogenesis occurs in the synovium and articular disc, leading to extracellular matrix degradation and subsequent cartilage destruction (34). In this process, matrix metalloproteinase 9 (MMP9) and vascular endothelial growth factor A1 (VEGFA) are key mediators of these changes. In subchondral bone, transforming growth factor-β1 (TGF-β1) and platelet-derived growth factor-BB (PDGF-BB) regulate angiogenesis. Consequently, targeting angiogenesis has shown promise in TMJOA treatment (35–37).

#### 4.2.2 Clinical manifestations

The clinical symptoms of TMJOA are diverse, including pain, limited jaw movement, and joint clicking. Currently, the diagnosis of TMJOA primarily relies on imaging techniques such as cone beam computed tomography (CBCT) and magnetic resonance imaging (MRI). Typical radiographic findings include erosive resorption, sclerosis, attrition, osteophyte formation, and cyst-like change (38–40). However, radiographic findings and patient symptoms may lag behind disease progression, meaning the initial stage of TMJOA can be subclinical (41). For early and precise identification of TMJOA, biomarkers derived from synovial fluid and serum have emerged as a promising approach (9, 42). For instance, E Kubota et al. (43) found that synovial fluid samples from TMJOA patients exhibited significantly higher levels of IL-6 and IL-1β compared to normal volunteers and patients with simple internal derangement (ID). These cytokines in the synovial fluid may serve as potential biochemical markers for cartilage degradation. Additionally, metabolomic and clinical examinations revealed a correlation between synovial fluid metabolites and TMJOA symptoms, suggesting their potential as biomarkers for grading disease severity (44). Notably, due to the invasiveness of arthrocentesis, researchers are exploring more convenient as well as noninvasive sources of biomarkers for analysis, such as saliva (45). With the aid of artificial intelligence, B Shoukri et al. (45) evaluated the correlation between saliva biomarkers and condylar morphology in TMJOA patients. They found that matrix metalloproteinase 3 (MMP-3), Vascular endothelial-cadherin (VE-cadherin), 6Ckine, and plasminogen activator inhibitor-1 (PAI-1) in saliva were significantly correlated with specific regions of condylar morphological variability. These findings highlight the potential of saliva-based biomarkers for early and accurate TMJOA diagnosis, warranting further exploration.

#### 4.2.3 Therapy and regeneration research

The primary objective of TMJOA therapy is to alleviate symptoms, halt disease progression, and restore the function of TMJ. Conventional treatment modalities for TMJOA extend from noninvasive management [e.g., physical therapy, occlusal splints, and nonsteroidal anti-inflammatory drugs (NSAIDs)] to invasive procedures such as arthrocentesis with lubrication (46). Notably, surgical interventions are usually considered last-option approaches in TMJOA therapy (20). Since severe destruction of condylar cartilage and extracellular matrix (ECM), as well as abnormal subchondral bone remodeling occur in the late stage of TMJOA, effective regeneration strategies are urgently needed. Recently, arthrocentesis with platelet-rich plasma (PRP), hyaluronic acid (HA), and mesenchymal stem cells (MSCs) was considered as a potential approach for regeneration (47–49). PRP was proven to enhance the proliferation of chondrocytes and MSCs (50, 51), and many growth factors such as TGF-β and platelet-derived growth factor (PDGF) in PRP contributed to this process (52, 53). Apart from its lubrication effect, HA was also found to have positive effects on cell growth as well as the chondrogenic differentiation of stem cells when combined with novel nanomaterials. For instance, Liu L et al. developed a hyaluronic acid-based microparticle that demonstrated significant cytoprotective and anti-inflammation effects on chondrocytes (54). Xiao, X et al. (55) also constructed a biomimetic tilapia type I gelatin/hyaluronic acid (TGI/HA) hydrogel, which can regulate the immune microenvironment and restore the damaged cartilage *in vivo*. Additionally, due to their potential for multi-lineage differentiation (including osteogenesis, adipogenesis, and chondrogenesis), MSCs gradually become a hotspot in the regeneration research of TMJOA. The use of various types of MSCs, including bone marrow mesenchymal stem cells (BMSCs) (56–58), human umbilical cord matrix-mesenchymal stem cells (hUCM-MSCs) (59), and adipose-derived stromal cells, (ASCs) (60) has been shown to exert positive effects on cartilage protection and regeneration. Notably, due to its rich enzymes and proteins, exosomes from MSCs also mediated the repair of TMJ, characterized by reduced inflammation and improvements of extracellular matrix and subchondral bone (61). Moreover, in TMJOA rats treated with exosomes from human embryonic mesenchymal stem cells, complete cartilage and subchondral bone restoration emerged 12 weeks after intra-articular injection (62). Therefore, a comprehensive exploration of the novel therapeutic potential of various stem cells could make significant progress for regeneration in TMJOA in the future.

### 4.3 Future research trends

According to the analysis above, predicting future trends and possible impacts on the search of TMJOA is significant. As illustrated in Figure 7B, the research directions have changed from “sodium hyaluronate”, “microarchitectural change”, “temporomandibular joint pathology”, “temporomandibular joint condylar morphology”, and “tmj osteoarthritis” to “critical signaling molecule”, “inhibiting chondrocyte ferroptosis”, “artificial intelligence”, “low-intensity pulsed ultrasound”, and “recent advance”, which could significantly influence future researchers. Notably, trend topic analysis (Figure 8B) identifies “oxidative stress”, “endoplasmic reticulum stress”, and “bone resorption regulation” as the most emergent research fronts, which also indicate that molecular mechanism elucidation represents the most promising research direction. Regarding pathological mechanisms, beyond classical chondrocyte apoptosis, emerging evidence highlights the critical involvement of novel programmed cell death modalities—particularly chondrocyte ferroptosis and pyroptosis—as breakthrough areas in TMJOA research. Ferroptosis of chondrocytes via multiple pathways provides novel insights into the pathogenesis of TMJOA, offering clinicians a more comprehensive understanding of its pathophysiological mechanisms. These findings also facilitate the development of targeted therapeutic strategies for TMJOA management. For instance, novel nanomaterials and traditional Chinese medicines targeting ferroptosis and pyroptosis of chondrocytes were found to alleviate the TMJOA. Specifically, mechanistic investigations have demonstrated that plumbagin, a bioactive naphthoquinone derivative isolated from *Plumbago* species (63–65), potently suppresses H2O2-induced chondrocyte ferroptosis through modulation of the MAPK signaling cascade (p38/JNK/ERK pathways), thereby mitigating TMJOA progression (66). Furthermore, emerging evidence demonstrated that poly (p-coumaric acid) nanoparticles (PCA NPs), as a novel nanotherapeutic platform, exhibit potent antioxidant and anti-inflammatory properties while effectively suppressing chondrocyte ferroptosis, thereby attenuating TMJOA progression (13). In addition, researchers found that resatorvid, a novel chemopreventive agent, alleviates TMJOA pathology by inhibiting chondrocyte pyroptosis and degeneration (67). These findings offer novel mechanistic insights into TMJOA pathogenesis, identifying potential therapeutic strategies for chondrocyte protection and regeneration, thereby guiding future translational research.

### 4.4 Strengths and limitations

This study offers a systematic overview of research hotspots and trends in TMJOA, providing actionable insights for researchers. However, several limitations warrant consideration. Firstly, reliance on the Web of Science Core Collection (WoSCC) database excluded studies from other platforms (e.g., PubMed, Cochrane, Embase), potentially introducing selection and publication bias. Furthermore, restricting inclusion to English-language articles and reviews may have omitted relevant non-English literature and non-article document types. In addition, the 2024 cutoff date for publication inclusion could exclude recent high-impact studies. Finally, the analysis did not evaluate citation context (e.g., supportive vs. critical citations) and may overlook smaller or interdisciplinary collaborations, which are often underrepresented in traditional bibliometric metrics.

## 5 Conclusion

This study employed a comprehensive bibliometric analysis utilizing CiteSpace, VOSviewer, Bibliometrix, and bibliometric web platforms to systematically evaluate publication trends, citation networks, and collaborative frameworks in TMJOA research. The analysis revealed distinct geographic leadership, with the United States and China emerging as predominant contributors to this field. These findings provide valuable guidance for oral and maxillofacial surgeons in identifying optimal publication venues and potential collaborative partnerships. Furthermore, keyword clustering identified four principal research domains: “pathogenic mechanisms”, “clinical manifestations”, “regeneration research”, and “therapy research”, offering actionable directions for future inquiry. Research trend analysis revealed a significant paradigm shift in TMJOA investigations, with an increasing focus on molecular signaling pathways and targeted therapies. Recent studies focused on elucidating chondrocyte ferroptosis regulation and other emerging programmed cell death mechanisms. Therefore, it is imperative to intensify investigations into these critical signaling molecules and their targeted therapies in TMJOA, with the ultimate goal of accelerating translational research to enhance clinical outcomes for patients. In conclusion, although significant progress has been made in TMJOA research, sustained and focused investigations remain imperative to generate novel mechanistic insights and therapeutic innovations that will ultimately improve human health.

## Data Availability

The original contributions presented in the study are included in the article/Supplementary Material, further inquiries can be directed to the corresponding author/s.
